# Incidence, outcomes and management of spontaneous haemoperitoneum in pregnancy: a UK population-based study

**DOI:** 10.3310/nihropenres.13960.2

**Published:** 2025-06-06

**Authors:** Ruth Tunn, Rema Ramakrishnan, Hilde Marie Engjom, Marian Knight

**Affiliations:** 1 National Perinatal Epidemiology Unit, University of Oxford, Old Road Campus, Headington, Oxford, OX3 7LF, UK; 2 Department of Health Promotion, Division Public Health and Prevention, Norwegian Institute of Public Health, Zander Kaaesgate 7, 5015 Bergen, Norway

**Keywords:** Spontaneous haemoperitoneum in pregnancy (SHiP), haemorrhage, pregnancy, incidence, surveillance, UK Obstetric Surveillance System (UKOSS), International Network of Obstetric Survey Systems (INOSS)

## Abstract

**Background:**

Spontaneous haemoperitoneum in pregnancy (SHiP) is the occurrence during pregnancy of sudden intra-abdominal haemorrhage unrelated to extrauterine pregnancy, trauma or uterine rupture. SHiP is uncommon but is associated with preterm birth, high perinatal mortality and, more rarely, maternal mortality. We investigated the incidence of SHiP in the UK and its diagnosis, management and outcomes.

**Methods:**

This two-year, prospective surveillance study used the UK Obstetric Surveillance System to collect anonymous data on all women who gave birth in a UK consultant-led maternity unit in 2016 and 2017 and who experienced SHiP.

**Results:**

We confirmed 20 cases of SHiP, giving an estimated incidence of 1.3 cases per 100,000 maternities, or 1 per 75,614 maternities. The median gestational age at diagnosis was 35.7 weeks (IQR 29.9–38.4 weeks). A minority of affected women were receiving anticoagulant agents for prophylaxis (2/20) or treatment (4/20). The most common initial suspected diagnosis was placental abruption (7/20), followed by intra-abdominal bleeding, uterine rupture, or infection. SHiP was diagnosed using ultrasound in four women, using CT in five, and solely at surgery in 14. Aneurysms (4/20) and organ rupture or haematoma (5/20) were the most common bleeding source, and the condition was most commonly diagnosed and treated by laparotomy (11/20). Perinatal morbidity and mortality were high, with 16% of infants stillborn, an over 80% admission rate to the neonatal unit among the 16 live-born infants, major complications in a third of these infants, and one neonatal death. Maternal morbidity was also high, with 60% of women admitted to the intensive care unit, over half of whom experienced major morbidity, and one maternal death.

**Conclusions:**

SHiP is rare in the UK but when it occurs, it can be associated with major maternal morbidity and mortality, and perinatal outcomes are poor. International comparisons are complicated by differing definitions of SHiP.

## Plain English summary

Spontaneous haemoperitoneum in pregnancy, or “SHiP”, is a rare but serious condition that can happen during pregnancy. It involves sudden internal bleeding into the belly that is not caused by an injury and does not come from the womb. We wanted to understand how common SHiP is in the UK, how women who experience it are diagnosed and treated, and what happens to them and their babies.

We worked with a national network of doctors and midwives who work in UK hospital maternity units to collect information for our study. They told us about every woman who gave birth in 2016 and 2017, and who experienced SHiP. In the two years of our study, about 1.5 million women gave birth in the UK. Of these, 20 women experienced SHiP – about one in 75,000. The most common symptom was belly pain. Over half of the women’s unborn babies’ heart rates were affected, and several women collapsed. A few women had an ultrasound or CT scan to diagnose the problem, but for most women, SHiP was diagnosed during surgery. Bleeding most often came from a burst blood vessel or damaged organ. SHiP was very dangerous for the women’s babies: three of the babies were stillborn and one died after birth. Most of the babies who survived needed to be cared for in the neonatal intensive care unit. SHiP was also very serious for the women themselves: one woman died, and over half needed treatment in the intensive care unit.

Our study showed that SHiP is very rare in the UK. However, when it does happen, in many cases it results in serious illness, and it can be fatal, for both mum and baby. Healthcare workers need to be alert for signs of SHiP.

## Introduction

Spontaneous haemoperitoneum in pregnancy (SHiP) is the occurrence during pregnancy of sudden intra-abdominal haemorrhage that is unrelated to extrauterine pregnancy, trauma, or uterine rupture. SHiP most commonly occurs in the third trimester but can occur at any time during pregnancy or in the weeks after birth ^1, 2^, and is associated with preterm birth, high perinatal mortality and, more rarely, maternal mortality ^1– 3^.

Known maternal risk factors for SHIP are maternal age over 35 years ^2^ and factors associated with endometriosis ^1, 4, 5^, including pregnancies conceived via artificial reproductive techniques (ART) ^6– 8^ as well as multiple pregnancy ^2^. Women experiencing SHiP typically present with sudden acute abdominal pain, frequently in combination with hypovolaemic shock, decreased haemoglobin levels, and/or signs of fetal distress ^7^. Bleeding may originate from abdominal parenchymal organs, abdominal arterial or venous vessels such as splenic artery aneurysms or pelvic varicose veins, from extrauterine endometroid or decidual tissue implants, or other causes ^1, 9, 10^. Often, placental abruption is initially suspected ^1, 7^. Diagnosis usually involves detection of free peritoneal fluid by ultrasound ^1^, sometimes in combination with other modalities such as computed tomography ^2, 3, 5^. Management is typically surgical, with laparotomy the most common intervention ^1, 2^, although expectant management was the most frequently used treatment approach in the Netherlands ^3^. The mode of birth is caesarean section in a majority of cases ^2, 3, 7^.

Because of the rarity of the condition, it is challenging to estimate the incidence of SHiP. Most reports of SHiP in the literature are individual case studies or small case series ^1^. The MBRRACE-UK surveillance programme identified six maternal deaths attributed to spontaneous intra-abdominal bleeding in the three years from 2019 to 2021 ^9^, but information on non-fatal case incidence and morbidity related to SHiP in the UK is lacking. In light of the evidence gap, International Network of Obstetric Survey Systems (INOSS) initiated a series of prospective nationwide surveillance studies in multiple countries ^11^.

A two-year study in the Netherlands found an estimated incidence of SHiP of 4.9 per 100,000 births, with no maternal mortality among 14 women, and three perinatal deaths ^3^. A similar study in Italy estimated the incidence at 4 per 100,000 births, with two maternal deaths, one neonatal death, and five stillbirths among 29 cases ^2^. Here, we present the findings of the UK Obstetric Surveillance System (UKOSS) study of SHiP.

The aim of this two-year, prospective, UK population-based surveillance study was to describe the incidence, diagnosis, and management of SHiP, and the characteristics and outcomes of affected women and their babies.

## Methods

### Patient and public involvement

Patients were not directly involved in the design of this study. Two members of the public were indirectly involved in the design of the study as representatives on the UKOSS Steering Committee, which reviews, comments on, and approves the choice of conditions to be investigated and the design and data collection forms for all studies to be run through UKOSS. Patients and the public were not involved in dissemination plans for the study.

### Study design and setting

For this national, two-year, prospective, population-based surveillance study the source population was 1,512,289 women giving birth in the UK from 2016 to 2017 ^12– 14^. Women were included if they had SHiP between 1 January 2016 and 31 December 2017. The UKOSS infrastructure was used for reporting and collection of clinical information; this methodology has been described in detail elsewhere ^15^. Briefly, nominated clinicians at all UK consultant-led maternity units complete a monthly return reporting the number of cases of specified uncommon conditions diagnosed in pregnant women by their managing clinicians (including zero values). Where a case is identified, UKOSS send a data collection form to gather data on the characteristics of the woman, her pregnancy, and the birth; potential risk factors for the specified condition; diagnosis and management of the condition; and maternal and neonatal outcomes. Data are double-entered into a study-specific database, with any queries directed back to the reporting centre. Where data collection forms are not returned, up to five reminders are sent to the reporting centre to minimise missing data.

### Case definition

Cases were defined as any woman at 20 weeks or more gestation with sudden intra-abdominal haemorrhage requiring surgery (CS, laparotomy, laparoscopy), without preceding trauma. Women with extrauterine pregnancy, trauma, or uterine rupture were excluded.

### Data collection

The study-specific data collected included information about maternal socio-demographic characteristics, known risk factors (history of endometriosis, prior abdominal surgery, treatment with anticoagulants), information about diagnosis and treatment of intraabdominal haemorrhage including the use of blood products, and maternal and neonatal outcomes. The full data collection form for this study can be found here: https://www.npeu.ox.ac.uk/assets/downloads/ukoss/forms/UKOSS-SHiP-v10---12-Apr-2016.pdf

### Statistical methods

We calculated the incidence of SHiP with 95% confidence interval (CI) using the total number of maternities in the UK in 2016 and 2017 as the denominator ^12– 14^. Demographic characteristics of the women and the diagnosis, management and outcomes of SHiP are summarised using descriptive statistics. Categorical data are presented as counts and as percentages of women for whom data were available for each variable; continuous data are summarized as medians with interquartile range (IQR). Analyses were conducted using StataNow MP 18.5 ^16^.

### Ethical approval

The UK Obstetric Surveillance System general methodology was approved by the London Multi-Centre Research Ethics Committee (04/MRE02/45; 24 September 2004) and ethics approval for the current study as a substantial amendment was granted by the North London REC1 (study reference 10/H0717/20, 19 August 2015). Consent was not required for the collection of anonymous routine data.

## Results

### Incidence of spontaneous haemoperitoneum in pregnancy

During the two years of the study, UKOSS representatives reported 27 women as having potential SHiP. Of these, seven did not meet the case definition, leaving 20 confirmed cases of SHiP. The total number of maternities in the UK in 2016 and 2017 was 1,512,289 ^12– 14^, giving an incidence of SHiP of 1.3 cases per 100,000 maternities (95% CI 0.81–2.24) or 1 case per 75,614 maternities.

### Demographic and clinical characteristics of women

Demographic and clinical characteristics of the 20 women are summarised in Table 1. The median age was 34.5 years (IQR 32–37 years). Two women had a history of endometriosis; four women had had a previous caesarean section, and seven had previous abdominal surgery of any kind. Two women had a known genetic bleeding tendency or haemoglobinopathy. Five women had a history of previous pregnancy problems; these included preeclampsia, intrauterine growth restriction, and placenta accreta.

**Table 1.** Demographic and clinical characteristics.

Characteristic	Total (N = 20)
**Maternal age (years), median (IQR)**	34.5 (32 – 37)
**Ethnic group, n (%)**	
White	15 (78.9)
Asian or Asian British	2 (10.5)
Black or Black British	2 (10.5)
*Missing*	*1*
**Employed, n (%)**	
No	7 (36.8)
Yes	12 (63.2)
*Missing*	*1*
**Body mass index at booking (kg/m ^2^),** **median (IQR)**	26.0 (23.4 –29.5)
**Smoking status during pregnancy, n (%)**	
Yes/gave up during pregnancy	3 (15.8)
No/gave up prior to pregnancy	16 (84.2)
*Missing*	*1*
**Problems in previous pregnancy/** **pregnancies, n (%)**	
No	8 (42.1)
Yes	5 (26.3)
Not applicable (nulliparous)	6 (31.6)
*Missing*	*1*
**Pre-existing medical problems, n (%)**	
No	10 (52.6)
Yes	9 (47.4)
*Missing*	*1*
**History of endometriosis, n (%)**	
No	17 (89.5)
Yes	2 (10.5)
*Missing*	*1*
Prior abdominal surgery *, n (%)	
No	12 (63.2)
Yes	7 (36.8)
*Missing*	*1*
**Previous caesarean birth, n (%)**	
No	15 (78.9)
Yes	4 (21.1)
*Missing*	*1*

*Including caesarean section

### Index pregnancy characteristics

Characteristics of the index pregnancy are summarised in Table 2. All were singleton pregnancies. Thirteen of the women had given birth previously. Prior to diagnosis of SHiP, the planned mode of birth was elective caesarean section in four women, vaginal in birth in fourteen, and missing in two.

**Table 2.** Index pregnancy characteristics.

Characteristic	Total n (%) (N = 20)
**Number of previous births**	
0	6 (31.6)
≥1	13 (68.4)
*Missing*	*1*
**Multiple pregnancy**	
No	19 (100.0)
Yes	0 (0.0)
*Missing*	*1*
**Planned mode of birth prior to diagnosis** **of SHiP**	
Vaginal (including trial of labour)	14 (77.8)
Abdominal (elective caesarean)	4 (22.2)
*Missing*	*2*
**Received anticoagulant during pregnancy**	
No	14 (70.0)
Yes	6 (30.0)
**Other problems during pregnancy**	
No	12 (60.0)
Yes	8 (40.0)

Problems reported during the index pregnancy included thrombotic events in three women, two women with HELLP and one woman with gestational diabetes.

Six women received anticoagulants during the index pregnancy: four received low molecular weight heparin (LMWH) only, one received acetylsalicylic acid, and one woman received both. LMWH was given prophylactically in one of these women and therapeutically in four. No other anticoagulant use was recorded.

### Diagnosis and management of spontaneous haemoperitoneum in pregnancy

Details of the diagnosis and management of SHiP are summarised in Table 3. The median gestational age at diagnosis was 35.7 weeks (IQR 29.9–38.4 weeks). Of the 20 women, 18 presented with abdominal pain. Other symptoms prior to diagnosis included abnormal fetal heart rate in over half of women (12/20, 60%); and less frequently, collapse, shoulder tip pain, altered uterine contractions, and vaginal bleeding. The most common initial suspected diagnosis was placental abruption (7/20, 35%), followed by intra-abdominal bleeding, uterine rupture, or infection. SHiP was diagnosed using ultrasound in four women, using CT in five, and solely at surgery in 14.

**Table 3.** Characteristics of SHiP.

Characteristic	Total (N = 20)
**Gestational age at diagnosis (weeks),** **median (IQR)**	35.7 (29.9 – 38.4)
*Missing*	*1*
Symptoms prior to SHiP diagnosis *	
Abdominal pain	18 (90.0)
Altered uterine contractions	2 (10.0)
Haematuria	1 (5.0)
Vaginal bleeding	2 (10.0)
Fetal heart rate abnormality	12 (60.0)
Shoulder tip pain	2 (10.0)
Collapse	4 (20.0)
Other	5 (25.0)
**Initial presumed diagnosis**	
Placental abruption	7 (35.0)
Intra-abdominal bleed	3 (15.0)
Uterine rupture	3 (15.0)
Infection	3 (15.0)
Other	4 (20.0)
Haemoperitoneum diagnosed *	
Peritoneal lavage	0 (0.0)
Ultrasound	4 (20.0)
CT	5 (25.0)
CTPA	0 (0.0)
MRI	0 (0.0)
Solely at surgery	14 (70.0)
**Mode of surgery for haemorrhage** **management**	
Laparoscopy	1 (5.3)
Planned ^†^ caesarean or hysterotomy	4 (21.1)
Laparotomy	11 (57.9)
Emergency ^‡^ caesarean or hysterotomy	3 (15.8)
*Missing*	*1*
**Signs of active endometriosis at** **surgery**	
No	15 (88.2)
Yes	2 (11.8)
*Missing*	*3*
**Site of pregnancy**	
Intrauterine	18 (100.0)
*Missing*	*2*
**Source of bleeding**	
Arterial aneurism ^§^	4 (20.0)
Organ rupture/haematoma ^¶^	5 (25.0)
Endometriosis	1 (5.0)
Other	7 (35.0)
None/none identified	3 (15.0)
**Estimated total blood loss (mL),** **median (IQR)**	3500 (2000 – 6000)
*Missing*	*2*
**Estimated intraperitoneal blood loss**	
<500ml	1 (5.0)
≥500 ml	19 (95.0)
Blood products given *	
Whole blood/packed red cells	18 (100.0)
Total units, median (IQR)	5 (4 – 15)
*Missing*	*2*
Fresh frozen plasma	12 (70.6)
Total units, median (IQR)	4 (1 – 7.5)
*Missing*	*3*
Platelets	12 (75.0)
Total units, median (IQR)	1.5 (1 – 2)
*Missing*	*4*
Cryoprecipitate	6 (46.2)
Total units, median (IQR)	2 (2 –5)
*Missing*	*7*
Cell salvaged blood	2 (20.0)
Total units (ml), median (IQR)	1399 (600 – 2198)
*Missing*	*10*
**Haemostatic drugs given**	
No	7 (35.0)
Yes *	13 (65.0)
Fibrinogen	1 (7.7)
Factor VII	3 (23.1)
Tranexamic acid	13 (100.0)

*More than one response possible ^†^Delivery of baby intended at onset of surgery ^‡^Delivery of baby not planned at the start of laparotomy ^§^Splenic or renal ^¶^Spleen or liver

The most common bleeding sources were aneurysms, rupture or hematoma in the spleen, liver or kidney (9/20 women, 45%). This was followed by pelvic sources associated with the internal genital tract and scar tissue after previous surgery. Only one woman had endometriosis as a source of bleeding.

Median blood loss was 3500 mL (IQR 2000–6000 mL). Around two-thirds of women (13/20) received haemostatic drugs, all of whom received tranexamic acid, with additional factor VIII and fibrinogen used in a minority of cases (n = 3 and n = 1, respectively). The most common mode of surgery for management of the haemorrhage was laparotomy, in 11 women (58%). Seven women (37%) had a caesarean or hysterotomy, which was planned from the start of the surgery in four women and followed conversion from laparotomy in three.

### Pregnancy outcomes

Twelve women (60%) were admitted to intensive care, with a median duration of stay of 3 days (IQR 1–10 days, data missing for one woman). Of these women, one had a miscarriage in the second trimester, and one died. Six women (30%) experienced major morbidity, including renal failure (n = 2), disseminated intravascular coagulation and multiorgan failure (n = 1) and cardiac arrest (n = 1). Two women required mechanical ventilation and two underwent a splenectomy.

### Perinatal outcomes

Perinatal outcomes are summarised in Table 4. Of the 19 women whose pregnancies proceeded beyond 24 weeks, three gave birth vaginally and 16 gave birth by caesarean section with urgency grade 1 (indicating immediate threat to life of woman or fetus; n=15, one of which was a resuscitative hysterotomy [perimortem CS]) or grade 2 (indicating maternal or fetal compromise not immediately life-threatening; n=1). The majority of caesarean sections (n=12) were performed under general anaesthesia. Indications for caesarean section included fetal bradycardia, distress or compromise (in nine women); placental abruption or suspected abruption (in three women); abdominal pain (in three women); and uterine rupture subsequent to SHiP management by laparotomy (in one woman).

**Table 4.** Perinatal outcomes.

Characteristic	Total (N = 19 * unless otherwise stated)
**Stillbirth**	
No	16 (84.2)
Yes	3 (15.8)
**Mode of birth**	
Spontaneous vaginal	2 (10.5)
Operative vaginal	1 (5.3)
Pre-labour caesarean	15 (78.9)
Caesarean after labour onset	1 (5.3)
**Induced labour**	
No	18 (100.0)
*Missing*	*1*
**Caesarean birth**	
No	3 (15.8)
Yes	16 (84.2)
**Grade of urgency (n = 16)**	
Immediate threat to life of woman or fetus	15 (93.8)
Maternal or fetal compromise not immediately life-threatening	1 (6.2)
**Method of anaesthesia (n = 16)**	
Regional	3 (20.0)
General	12 (80.0)
*Missing*	*1*
**Placental abnormality identified**	
No	17 (94.4)
Yes	1 (5.6)
*Missing*	*1*
**Birthweight (g)**, median (IQR)	2855 (1322 – 3485)
*Missing*	*1*
**Apgar @5 minutes**, median (IQR) **(n = 16)**	6 (4 – 9)
*Missing*	*1*
**Umbilical artery pH (n = 16)**	
Not measured	4 (28.6)
Measured	10 (71.4)
Median (IQR) pH	6.9 (6.8 –7.0)
*Missing*	*2*
**Neonatal unit admission (n = 16)**	
No	3 (18.8)
Yes	13 (81.3)
**Major infant complications (n = 16)**	
No	10 (66.7)
Yes	5 (33.3)
*Missing*	*1*
**Neonatal** **death (n = 16)**	
No	14 (93.3)
Yes	1 (6.7)
*Missing*	*1*

*One woman miscarried

The median birthweight of the 19 infants born at or after 24 weeks was 2855g (IQR 1322–3485g). There were three recorded stillbirths. Among live-born infants, the median 5-minute Apgar score was 6 (IQR 4–9). The median umbilical artery pH was 6.9 (IQR 6.8–7.0) in the ten infants for whom this information was recorded. Thirteen infants were admitted to the neonatal intensive care unit, with five experiencing major complications that included respiratory distress syndrome, hypoxia, jaundice, hypokalaemia, and suspected sepsis. One infant subsequently died of hypoxic-ischemic encephalopathy.

## Discussion

This two-year, prospective, UK population-based surveillance study estimated the incidence of SHiP as 1.3 cases per 100,000 maternities, equating to 1 case per 75,614 maternities. Aneurysms and rupture or haematoma in the liver, spleen or kidney were the most common sources of bleeding. The diagnosis was most commonly made and treated during open abdominal surgery. A minority of affected women were receiving anticoagulant agents for prophylaxis (2/20) or treatment (4/20) of thromboembolism. Severe maternal and perinatal outcomes were common; these included stillbirths, neonatal intensive care unit admission, maternal intensive care unit admission, and one maternal death.

This estimated incidence of SHiP is considerably lower than that estimated using similar national surveillance systems in the Netherlands (4.9 cases per 100,000 births) and Italy (4 cases per 100,000 births). All countries collaborate in the INOSS network and based their study on a joint definition of SHIP. However, local adaptations to this definition need to be considered in interpretation of differences across countries. The Dutch study ultimately deviated from the INOSS definition of SHiP ^11^ and decided to exclude abdominal bleeding from aneurysms or liver, spleen and kidney. After these exclusions the management was expectant in over a third of included women, while fewer than two thirds underwent either surgery (6/14) or embolisation (2/14) ^3^. Our study also deviated from the INOSS definition, but by including only cases requiring surgery (rather than those requiring either surgery or embolisation), which could have led us to underestimate incidence. Management by endovascular intervention depends on availability of expertise, including in interventional radiology, in the hospital. Our inclusion criteria may account for the some of the disparity between our findings and those of the Italian surveillance ^2^, which used the INOSS definition, although the authors do not report the number of women who were eligible for inclusion as a result of having undergone embolisation.

Subjectivity of diagnosis may also be a contributing factor in the differing estimates of incidence – in the Dutch study, auditors disagreed initially as to whether a SHiP diagnosis was appropriate in almost half (9/20) of potential cases submitted by reporters. There may also be underlying differences between the three countries in the risk profile of the pregnant population: for example, ART, an established risk factor for SHiP ^6, 7^, contributes to 4.2% of births in Italy ^17^ and 3.0% in the Netherlands ^18^ versus 1.2% in the UK ^19^. Unfortunately, we did not explicitly request data on ART in our study, although IVF was mentioned in the supplementary notes of one woman; five of the 14 women in the Dutch study and five of the 29 in the Italian study had conceived by ART.

We found that aneurysms, rupture or hematoma in the spleen, liver or kidney were the most common causes of SHiP. Pregnancy is associated with vascular wall and hemodynamic changes, and also increases the risk of haemorrhage/rupture of intracerebral and aortic aneurysms ^20– 22^. Vascular changes and hypertension in preeclampsia may aggravate this, but only two women in our study had pre-existing hypertensive disorders during the index pregnancy. Only one woman had endometriosis as the source of bleeding; this low frequency is striking even when exempting from the denominator cases arising from liver hematoma/rupture known to be associated with hypertensive disorders of pregnancy and HELLP-syndrome. By contrast, endometriosis was present in around a fifth of women with SHiP identified by the Italian surveillance ^2^ and a third of women identified in the Netherlands ^3^.

SHiP was diagnosed by imaging prior to surgery in fewer than half of the women in our study: five of 20 were diagnosed using CT scan, four using ultrasound and none by MRI. This is in contrast to diagnosis in the Netherlands, where all affected women in a 2-year surveillance study underwent ultrasound, around a third (4/13) underwent CT, and a third (4/13) underwent MRI. This likely reflects the higher proportion of hemodynamically stable women included under the Dutch definition of SHiP, whereas CT or MRI is unlikely to be appropriate in the more emergent cases captured by the case definition in our study. The diagnostic modalities in Italy more closely resembled that in the UK: 14 of 29 women underwent ultrasound, six underwent CT and none MRI. Focused assessment sonography for trauma (FAST), used effectively in non-pregnant trauma patients with suspected haemoperitoneum ^23^, offers a more rapid alternative to MRI or CT that may be more appropriate for diagnosing SHiP in haemodynamically unstable women.

Almost a third of women in our study (6/20) were receiving anticoagulant agents, and only four were receiving therapeutic high-dose LMWH for thrombosis or thromboembolism. Thrombosis and thromboembolism are now the leading cause of maternal deaths in the UK during pregnancy or in the six weeks following birth ^24^. Royal College of Obstetricians and Gynaecologists guidelines recommend prophylactic LMWH during pregnancy for women with previous venous thromboembolism or certain combinations of risk factors ^25^, and LMWH treatment in clinically suspected pulmonary embolism or deep vein thrombosis ^26^, unless strongly contraindicated. Awareness of the potential for SHiP, albeit rare, in these women, is important.

## Conclusion

SHiP is rare in the UK but when it does occur, it can be associated with major maternal morbidity and mortality, and perinatal outcomes are poor. Healthcare providers should be aware of the possible range of symptoms and consider it as a possible alternative diagnosis to placental abruption, intra-abdominal pain, uterine rupture, or abdominal infection, particularly in pregnant women presenting with abdominal pain and/or circulatory compromise. Future research would benefit from a strengthened consensus on the definition of SHiP.

## Data Availability

Data cannot be shared openly because of confidentiality issues and the potential identifiability of sensitive data. Requests to access the data can be made by contacting the National Perinatal Epidemiology Unit data access committee via general@npeu.ox.ac.uk. The Research Ethics Committee approved the study on the basis that access will only be allowed after review of the request by the UK Obstetric Surveillance System Steering Committee. The estimated response time for requests is 4 weeks. Data sharing outside the UK or European Union may require consultation with the UK Health Research Authority. For more information, please refer to the National Perinatal Epidemiology Unit Data Sharing Policy available at https://www.npeu.ox.ac.uk/assets/downloads/npeu/policies/Data_Sharing_Policy.pdf. For the purpose of open access, the authors have applied a Creative Commons Attribution (CC BY) licence to any Author Accepted Manuscript version arising.

## Software availability statement

All statistical analyses were performed using StataNow MP v18.5, a commercial software. All analyses in this study can be replicated using freely available statistical software such as R.
