# Supplementary material for: Incidence, outcomes and management of spontaneous haemoperitoneum in pregnancy: a UK population-based study
Source: NIHR Open Res. 2025 Jun 6;5:40. Originally published 2025 Apr 28. [Version 2] doi: 10.3310/nihropenres.13960.2 (PMC12198727; doi:10.3310/nihropenres.13960.2)
Supplement: Peer review history

# Reviewer report: Reviewer response for version 2

**DOI:** 10.3310/nihropenres.15224.r35956

**Published:** 2025-06-25

**Authors:** Johnbosco E. Mamah

**Affiliations:** 1 University of Nigeria, Nsukka, Enugu, Nigeria

No further comment

Is the work clearly and accurately presented and does it cite the current literature?

Yes

If applicable, is the statistical analysis and its interpretation appropriate?

Yes

Are all the source data underlying the results available to ensure full reproducibility?

Yes

Is the study design appropriate and is the work technically sound?

Yes

Are the conclusions drawn adequately supported by the results?

Yes

Are sufficient details of methods and analysis provided to allow replication by others?

Yes

Reviewer Expertise:

Benign gynaecology

I confirm that I have read this submission and believe that I have an appropriate level of expertise to confirm that it is of an acceptable scientific standard.

# Reviewer report: Reviewer response for version 1

**DOI:** 10.3310/nihropenres.15176.r35752

**Published:** 2025-06-03

**Authors:** Julia Sanders

**Affiliations:** 1 Cardiff University, Cardiff, UK

The paper presents results of a study to explore the incidence and outcomes of Spontaneous Hemoperitoneum in Pregnancy (SHiP) using the established UK Obstetric Surveillance System. 

 A total of 20 cases meeting eligibility definitions were reported across UK obstetric units in the years 2016 and 2017, giving an incidence of 1 per 75,614 maternities.

 The characteristics of women and the outcomes for women and their babies are reported.

 One maternal death was attributable to SHiP in 2016 and 2017, compared to six maternal deaths attributable to SHiP in the MBRRACE-UK surveillance programme during the three years from 2019 to 2021. It cannot be determined if the differences across these time periods was due to underreporting in the UKOSS study or reflected normal variation of a rare event.

 Median blood loss was 3500ml with sources of bleeding including arterial aneurysm (n=4), organ rupture (n=5) and endometriosis (n=1).

 Despite the severity of SHIP no particular risk factors were sufficiently evident to inform clinical practice. 

 In the event of severe abdominal pain in pregnancy, in the absence of a confirmed alternative diagnosis, clinicians need to remain aware of the potential for this alternative but rare cause of abdominal bleeding, and all obstetric units need facilities and access to trained staff to deal with surgically complex massive haemorrhage during pregnancy.

Is the work clearly and accurately presented and does it cite the current literature?

Yes

If applicable, is the statistical analysis and its interpretation appropriate?

Yes

Are all the source data underlying the results available to ensure full reproducibility?

Yes

Is the study design appropriate and is the work technically sound?

Yes

Are the conclusions drawn adequately supported by the results?

Yes

Are sufficient details of methods and analysis provided to allow replication by others?

Yes

Reviewer Expertise:

Midwifery and Maternity

I confirm that I have read this submission and believe that I have an appropriate level of expertise to confirm that it is of an acceptable scientific standard.

## References

[1] : Incidence, outcomes and management of spontaneous haemoperitoneum in pregnancy: a UK population-based study. *NIHR Open Research* .2025;5: 10.3310/nihropenres.13960.1 10.3310/nihropenres.13960.1 PMC1219872740575467

# Reviewer report: Reviewer response for version 1

**DOI:** 10.3310/nihropenres.15176.r35564

**Published:** 2025-05-26

**Authors:** Johnbosco E. Mamah

**Affiliations:** 1 University of Nigeria, Nsukka, Enugu, Nigeria

It was a well-written study. Why has it taken over 8 years since the end of data collection to index this work? Since the authors had such a long intervening time, why was data collection not extended? There is much literature on this subject from the UK between 2017 and now. Including this data would have added to the strength of this study. These recent studies show endometriosis to be a common risk factor among patients who have suffered SHiP. More women are being diagnosed with endometrosis in the last 5 years due to more campaigns and awareness, and so that's a huge data point missing in this study.

Is the work clearly and accurately presented and does it cite the current literature?

Yes

If applicable, is the statistical analysis and its interpretation appropriate?

Yes

Are all the source data underlying the results available to ensure full reproducibility?

Yes

Is the study design appropriate and is the work technically sound?

Yes

Are the conclusions drawn adequately supported by the results?

Yes

Are sufficient details of methods and analysis provided to allow replication by others?

Yes

Reviewer Expertise:

Benign gynaecology

I confirm that I have read this submission and believe that I have an appropriate level of expertise to confirm that it is of an acceptable scientific standard, however I have significant reservations, as outlined above.
